# Preparation and Properties of Antibacterial Polydopamine and Nano-Hydroxyapatite Modified Polyethylene Terephthalate Artificial Ligament

**DOI:** 10.3389/fbioe.2021.630745

**Published:** 2021-03-31

**Authors:** Yang Wu, Yuhan Zhang, Ren Zhang, Shiyi Chen

**Affiliations:** ^1^Department oft of Orthopaedic Sports Medicine, Huashan Hospital Affiliated to Fudan University, Shanghai, China; ^2^Center for Analysis and Measurement, Fudan University, Shanghai, China

**Keywords:** polydopamine, nano-hydroxyapatite, PET, anterior cruciate ligament, antibacterial, artificial ligament

## Abstract

Due to its great biomechanical property, the polyethylene terephthalate (PET) artificial ligament has become one of the most promising allografts for anterior cruciate ligament (ACL) reconstruction. However, because of its chemical and biological inertness, PET is not a favored scaffold material for osteoblast growth, which promotes the ligament-bone healing. Meanwhile, in consideration of prevention of potential infection, the prophylactic injection of antibiotic was used as a post-operative standard procedure but also has the increasing risk of bacterial resistance. To face these two contradictions, in this article we coated a polydopamine (PDA) nano-layer on the PET ligament and used the coating as the adhesion interlayer to introduce nano-hydroxyapatite (nHA) and silver atoms to the surface of PET ligament. Because of the mild self-polymerization reaction of dopamine, the thermogravity analysis (TGA), Raman spectrum, and tensile test results show that the modification procedure have no negative effects on the chemical stability and mechanical properties of the PET. The results of NIH3T3 cell culture show that the PDA and nHA could effectively improve the biocompatibility of PET artificial ligament for fibroblast growth, and staphylococcus aureus antibacterial test results show that the Ag atom provided an antibacterial effect for PET ligament. As shown in this paper, the nano-PDA coating modification procedure could not only preserve the advantages of PET but also introduce new performance characteristics to PET, which opens the door for further functionalization of PET artificial ligament for its advanced development and application.

## Introduction

The rupture of the anterior cruciate ligament (ACL) is a serious surgical injury, especially with the increasing participation of extreme exercise and high-intensity exercise in recent years (Monk et al., 2016). The rupture of anterior cruciate ligament makes the patient unable to control the knee joint and seriously affect the quality of life. In order to treat this injury, artificial ligaments are usually used to replace broken ligaments in surgery to reconstruct the patient’s control of the knee joint (Lai et al., 2018). At present, an artificial polymer artificial ligament based on a polyethylene terephthalate (PET) bundle, with high mechanical strength, high chemical inertness, and no biological toxicity is commonly used as an artificial ligament in anterior cruciate ligament reconstruction (ACLR). However, PET artificial ligament cannot effectively promote bone healing and patient recovery, which might lead to tunnel enlargement and delay recovery of patients because of its chemical inertia (Gao et al., 2010). To overcome this defect, many research groups have developed different protocols, such as chemical coating (Ding et al., 2019), mechanical mixing, and so on. For the chemical inertness of PET, the preceding methods may lead to ineffectively attachment of modified layer and even fall off. Therefore, it is necessary to find a modified method with good biocompatibility, strong adhesion, and easy operation.

On the other hand, intraoperative infection during ACLR has serious consequences and might induce infectious osteomyelitis (Ridley et al., 2018). As a routine, intravenous injection of broad-spectrum antibiotics for patients is wildly recommended to avoid infection, which however brings the risk of antibiotic abuse. If the precise antibacterial in the surgical site can be achieved, it can not only effectively avoid infection but also avoid the abuse of antibiotics. Therefore, we hope to achieve this goal through certain antibacterial modification of PET ligament.

Dopamine (DA) has a similar structure with dihydroxyphenylalanine (DOPA) contained in the mucin secreted by mussels, such as catechol functional groups and lysine end amino groups, which have been proved to have high adhesion properties. DA can be oxidized and self-polymerized in alkaline environment, forming a strong sticky polydopamine (PDA) layer on the surface of the material, thus achieving the coating on the surface of various materials (Zhang et al., 2016). As the self-polymerization of DA has the advantages, such as convenient preparation, environment-friendship reaction, and high-efficiency process, PDA have attracted more and more researchers to participate in the its application (Ryu et al., 2010; Shi et al., 2014). The research results show that the modification of the PDA-modified method can significantly improve the biological and pharmaceutical properties of the materials, and the good application prospect of the modified PDA method.

Hydroxyapatite is the main component of vertebrate bones and teeth, which has good bioactivity and biocompatibility. Hydroxyapatite not only has strong corrosion resistance but also strong osteoinduction (Bose and Tarafder, 2012). At present, the research on hydroxyapatite in bone substitute materials mainly focuses on hydroxyapatite coating and human bone biomimetic regeneration materials (Szczes et al., 2017). Meanwhile, nano-hydroxyapatite (nHA) has larger specific surface area and better biological properties than hydroxyapatite because of its nano effect; therefore, there have been reports on osteoinduction of nHA in recent decades (Zhou and Lee, 2011). In this study, nHA was coated on the surface of the PET artificial ligament for the purpose of promoting its biocompatibility and improving osseointegration after ACLR.

Silver has been used as a broad antibacterial material for thousands of years. Silver ions could bond with protein through the sulfur/nitrogen/oxygen atom on the amino acid side chain, which causes the destruction of cell membrane function and leads to the inhibition of bacterial growth (Lemire et al., 2013). In recent years, the silver antibacterial medical products, such as silver ion dressing, have been certified by FDA and used in surgery (Chernousova and Epple, 2013), and silver nano-particles were used in antibacterial modification of PET artificial ligament (Wang K. et al., 2020). Antibacterial ability of silver has following characteristics, such as broad-spectrum and sustained release, which make silver good candidate for antibacterial materials of artificial ligament.

**FIGURE 1:**
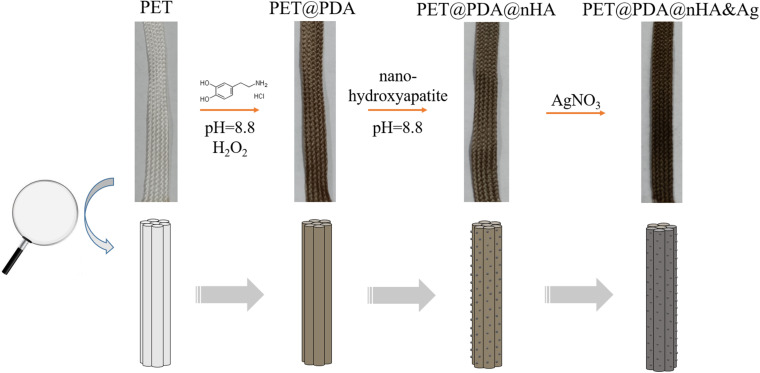
The schematic diagram of the PET ligament modification process. Up line is the photo images of ligaments, and down line is morphology change diagram of ligament fibers.

In this paper, we modified nHA and silver atoms on the surface of PET artificial ligament through the PDA layer without damaging the mechanical properties of PET. There are three steps as shown in Figure 1. First step: A layer of DA was modified on the surface of PET artificial ligament by self-polymerization of DA hydrochloride in alkaline and weak oxidation environment, which is expected to improve the surface properties of the PET artificial ligaments, such as biocompatibility and modifiable. Second step: in order to verify the modification of the PET artificial ligaments in the PDA layer, this article will also use the adhesion properties of PDA to modify the nHA on the surface of the PET artificial ligament, which may improve the cell proliferation of the PET artificial ligament. Third step: the silver nitrate reacts with PDA and nHA on the PET surface to generate silver compounds and provide PET ligament the antibacterial ability.

## Experiment

### Materials

DA hydrochloride, Tris(hydroxymethyl)aminomethane (Tris), hydrochloric acid (HCl), copper sulfate (CuSO_4_), and 30% hydrogen peroxide (H_2_O_2_) were purchased from Sigma-Aldrich, United States. The nHA was purchased from Aladdin reagent, China. The other reagents were purchased from Sinopharm Chemical Reagent limited corporation, China. The clinical PET artificial ligament was purchased from Shanghai Kinetic Co., Ltd. (KMC).

### Characterization

The scanning electron microscope image was scanned by Phenom Prox (Phenom, Holland) after Conductive Coating with gold on samples. Raman spectrum was recorded by XploRA Raman spectrometer (Horiba Jobin Yvon, France) with an 80mW 785nm wavelength excitation laser. The thermogravimetric analysis (TGA) was recorder by Pyris 1 TGA (Perkinelmer, United States). Tensile testing was tested by Instron 5966 Universal Testing Systems (Instron, United States). Elemental analysis was tested by PerkimElmer Optima 8000DV.

### Modification of Polydopamine and nHA

10 cm-length PET artificial ligament was placed in a 15 mL plastic centrifuge tube, then 10 mL 50 mM pH8.8 Tris-HCl buffer solution, 25 uL 1M CuSO_4_, 100 uL 30% H_2_O_2_ and DA were added to the tube, respectively. After mixing the reaction solution completely, the tube was placed in 37°C water-bath for 6 h. Finally, the PET ligament was washed by deionized water for 3 times and dried in 25°C ovens. The prepared PDA-modified PET ligament (PET@PDA ligament) was placed in a 15 mL plastic centrifuge tube, then 10 mL 50 mM pH8.8 Tris-HCl buffer solution, 25 μL 1 M CuSO_4_, 100 μL 30% H_2_O_2_, 20 mg DA and 100 mg nHA were added to tube, respectively. The nHA reaction solution was mixed by rotary mixer for 2 h. Then the nHA modified PET@PDA ligament (PET@PDA@nHA ligament) was dried in 25°C ovens.

### Modification of Silver Atoms

A 10 cm PET, PDA-modified, PDA- and nHA-modified PET artificial ligament was placed in a 15 mL plastic centrifuge tube, then 15 mL 100 mg silver nitrate contained aqueous solution was added. The solution was placed on rotary blender for 2 h. Then the ligament was washed five times. The abbreviation of the above three Ag modified ligaments was PET@Ag, PET@PDA@Ag and PET@PDA@nHA&Ag, respectively.

### The Antibacterial Performance

Staphylococcus aureus (SA, CMCC26003) was cultured in LB medium until the OD600 value was about 0.6, which was corresponding to 108 CFU/mL concentrations. The bacterial solution was diluted to 10^5^ CFU/mL at the dilution ratio of 1:1,000. The tested samples were placed in 2 mL bacterial solution at 37°C for 24 h to detect the optical density (OD) value.

### Cell Proliferation to NIH3T3 and MG-63 Cells

To evaluate the cytotoxicity and osseointegration of the modified ligaments, the metabolic viability of cells cultured with the ligaments (one 5 mm-long fiber of ligament) was measured using the Cell Counting Kit-8 (CCK-8) assay on mouse embryonic fibroblast cell line (NIH3T3) and human osteosarcoma cell line (MG-63). First, the NIH3T3 and MG-63 cells were seeded in a 96-well tissue culture plate at a seeding density of 10,000 cells/well for 24 h, respectively. After the cell adheres to the wall, the ligaments were clamped into the pore plate. Then, after 72 h of incubation, the solution was removed from the well plates. PBS was used to wash the ligaments for two times, then add 1 mL 10% CCK8 medium and continue culturing for 2 h. Finally, the solution was removed into a 96-pore ELISA plate for testing.

## Results and Discussion

### The Modification of PDA

In alkaline and oxidizing atmosphere, the hydroxyl group of DA is oxidized to quinone structure. Then the quinone type DA was polymerized through the carbon atoms on the benzene ring, resulting in the formation of DA (Liu et al., 2014). The polymerization of DA can be observed on the macro level, usually manifested by obvious color changes in the substrate material involved in the polymerization of DA. Similar to the color changes in literature (Zhang et al., 2016), PET artificial ligaments are transformed from white to gray black (Figure 1) after being modified by PDA, indicating that the PDA has been modified on the surface of the PET. It is worth noting that, from the color distribution of the PDA-modified PET artificial ligaments, the modification of PDA is uniform, indicating that the modification method of the PDA can be applied to the large batch preparation of the artificial ligaments of the PET@PDA.

**FIGURE 2:**
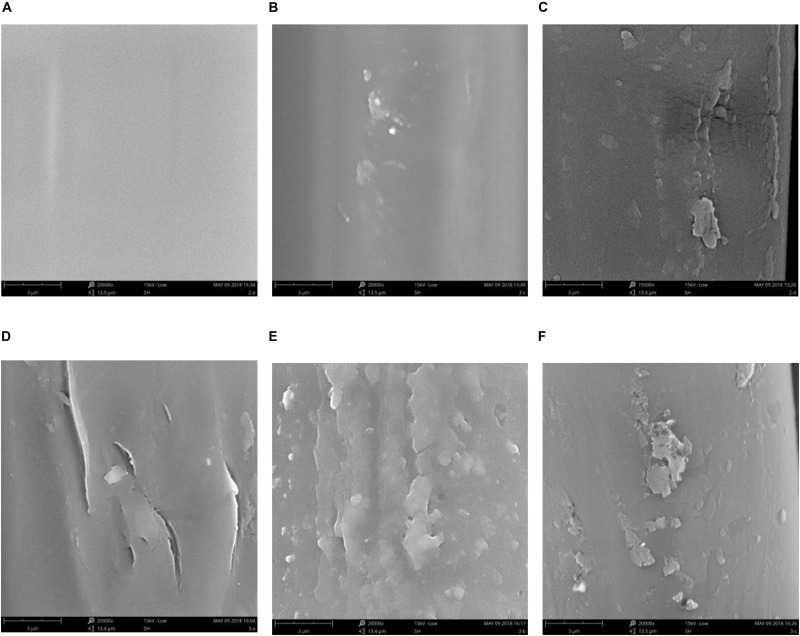
Surface SEM images of different DA-concentration-modified PET ligament. **(A)** 0 mg, **(B)** 5 mg, **(C)** 10 mg, **(D)** 20 mg, **(E)** 50 mg, and **(F)** 100 mg.

To investigate the formation process of PDA layer, a series of different amount of DA-modified PET ligament was processed, and the SEM images was shown in Figure 2. As Figure 2 shows, the surface of pure PET (Figure 2A, 0 mg DA) is smooth, and there is no significant foreign body on it. When the amount of DA is up than 5 mg, the significant roughened particles could be observed, which may indicate the growth process of PDA on the substrate surface. When the DA amount is up to 20 mg, the continuous PDA layer is formed as shown in Figure 2D, when DA amount continue increasing, the roughness is also increasing (Figures 2E,F). The SEM results show that the PDA layer is formed from the distributed particles at the beginning of the PDA layer by the increasing of the DA amount.

**FIGURE 3:**
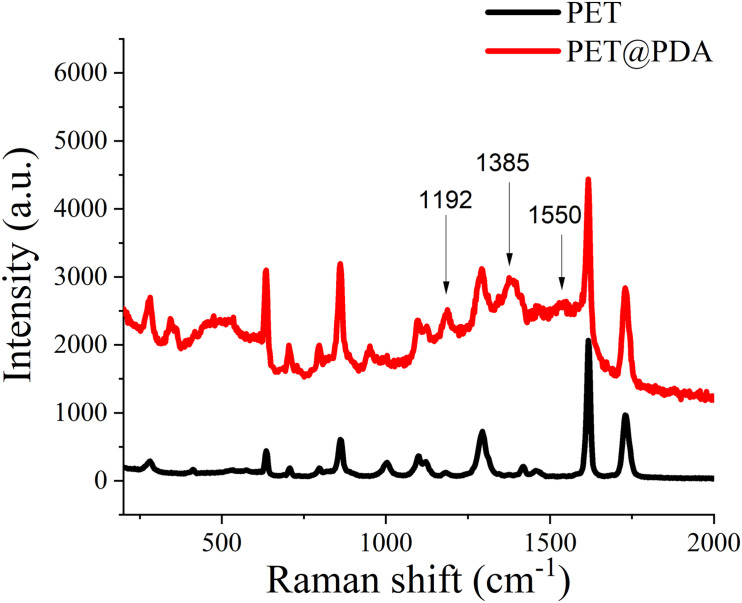
Raman spectrum of PET artificial ligament (black) and PDA-modified PET artificial ligament (red).

Raman spectroscopy is a non-destructive and sensitive surface analysis method, which can identify the structure or composition of the material surface by the vibration spectrum of the material. By analyzing the Raman spectra of PET and PET@PDA artificial ligaments, the adhesion of PDA on PET can be verified structurally. As shown in Figure 3, the Raman spectra of PET@PDA have 1192, 1,385 and 1,550 cm^–1^ scattering peaks, which is absent in the PET’s spectrum, and the three peaks are were contributed from the PDA signal, which originate from the vibration of aromatic rings and aliphatic C–C and C–O stretching, respectively. Meanwhile, the baseline of PET is flat, but the baseline of PET@PDA has apparent fluctuation, which is contributed from PDA, which is originated from the complexity of PDA’s structure (Li et al., 2018).

**FIGURE 4:**
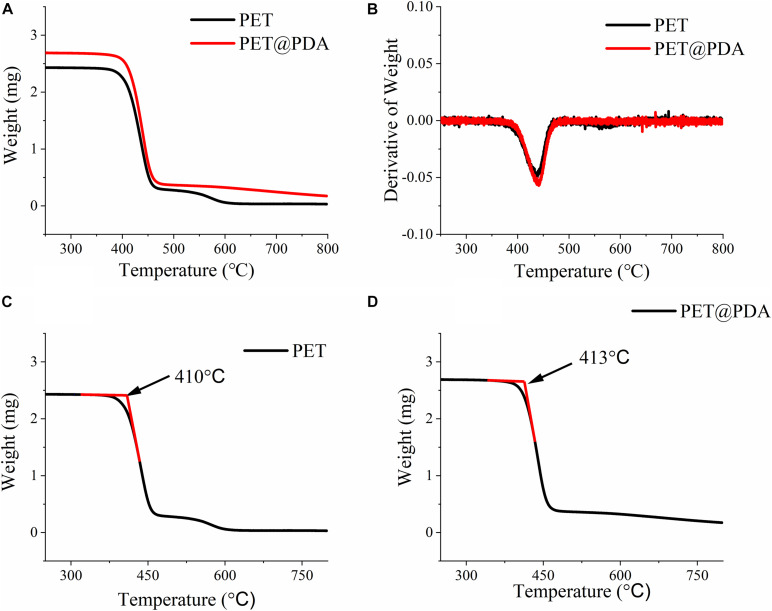
Thermal gravity curve **(A)** and differential thermal gravity curve **(B)** of PET and PET@PDA ligament. Onset decomposition temperature of PET **(C)** and PET@PDA **(D)** ligament.

In order to verify whether the main structure of PET was damaged after DA modification, the thermogravimetric analysis was used. TGA could reflect the thermal stability and composition of materials by studying the mass loss of materials during the heating process. Figure 4A is the thermogravimetric curve of PET and PET@PDA. In the chart, the two materials have shown the similar weight-loss curves. Differential thermal gravity (DTG) curves of two materials can be obtained by differential treatment of thermogravimetric curves, as shown in Figure 4B. The DTG curves show that the weight-loss rates of the two materials are almost the same, indicating that the thermal stability of PET has not changed after modification with PDA. On the other hand, by analyzing the onset decomposition temperature (Figures 4C,D), it is found that the temperatures of both materials are about 410°C. The data of DTG and onset decomposition temperature show that the thermal stability of PET has not changed after PDA modification, indicating the modification of PDA has not changed the chemical properties of the material. On the other hand, no signal of PDA was observed in DTG, which may be due to the insufficient content of PDA on the PET surface to form a signal in the DTG curve, which also indicates that the PDA modification method is an accurate and nano-scale surface modification method.

### The Modification of nHA

**FIGURE 5:**
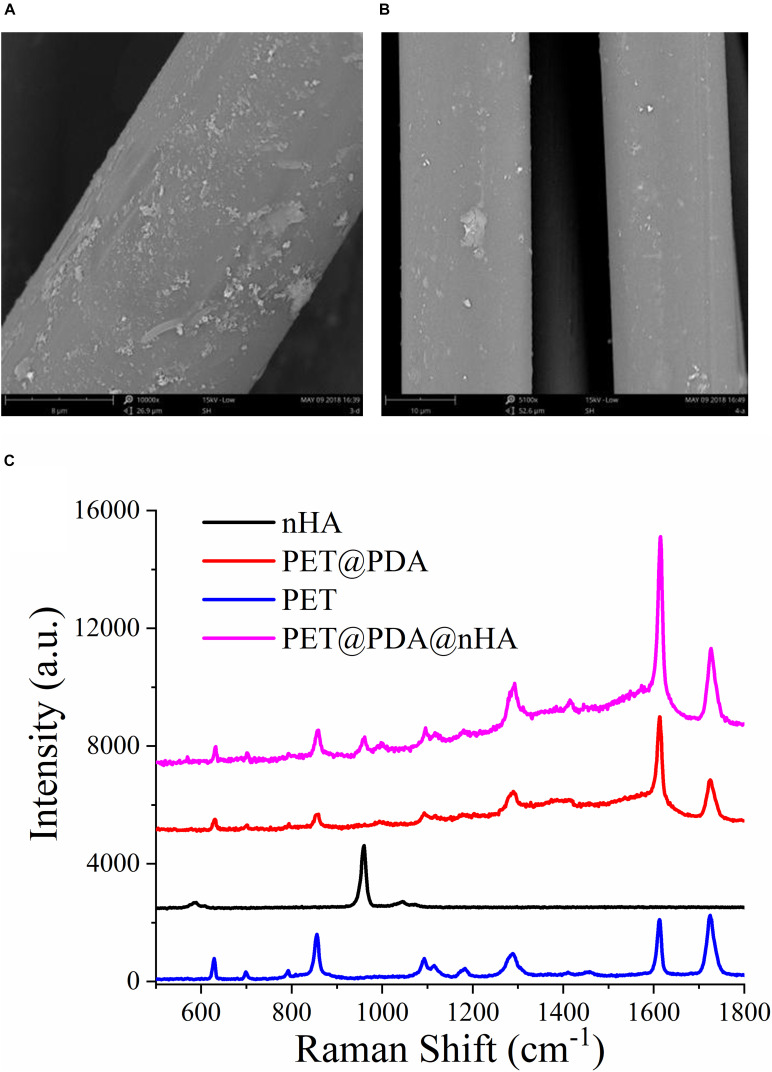
SEM images of PET@PDA@nHA, which were modified by two steps **(A)** and one step **(B)** reaction, and Raman spectra of PET and PET@PDA@nHA **(C)**.

PDA not only can modify the surface of PET but also can be used as an adhesive layer to bond other functional materials to the surface of PET. nHA is widely used in the field of bone growth, bone healing, and so on as a surface component of bone structure (Sadat-Shojai et al., 2013). In order to promote the bone growth and healing of the PET ligament and bone junction, we use the PDA on the PET fiber as the adhesive layer and adhere nHA to the PET ligament, thus preparing the hydroxyapatite-modified PET@PDA ligament (PET@PDA@nHA). There are two methods to prepare PET@PDA@nHA. One is to adhere to the PDA after the preparation of the PET@PDA (two-step), and the other is to add nHA in the DA polymerization process (one-step). Figure 5 is the SEM image of the two methods followed by nHA, in which the Figure 5A is the two-step method, and the Figure 5B is the one-step method of PET@PDA@nHA. It can be seen from the diagram that there are obvious differences between the two modified materials, in which the two-step modified PET@PDA@nHA fibers have more nHA on the surface than the one-step method. The results of Raman spectra also show the same results. From Figure 5C, it could be seen that the two-step modified fibers can see the more obvious Raman signal of nHA, while the one-step method can hardly see the signal of nHA. The results of SEM and Raman spectra indicated that PDA did play a role in the functional material adhesion layer, and the two-step modification of PET fibers had more efficient modification efficiency.

### The Modification of Silver Atoms

**FIGURE 6:**
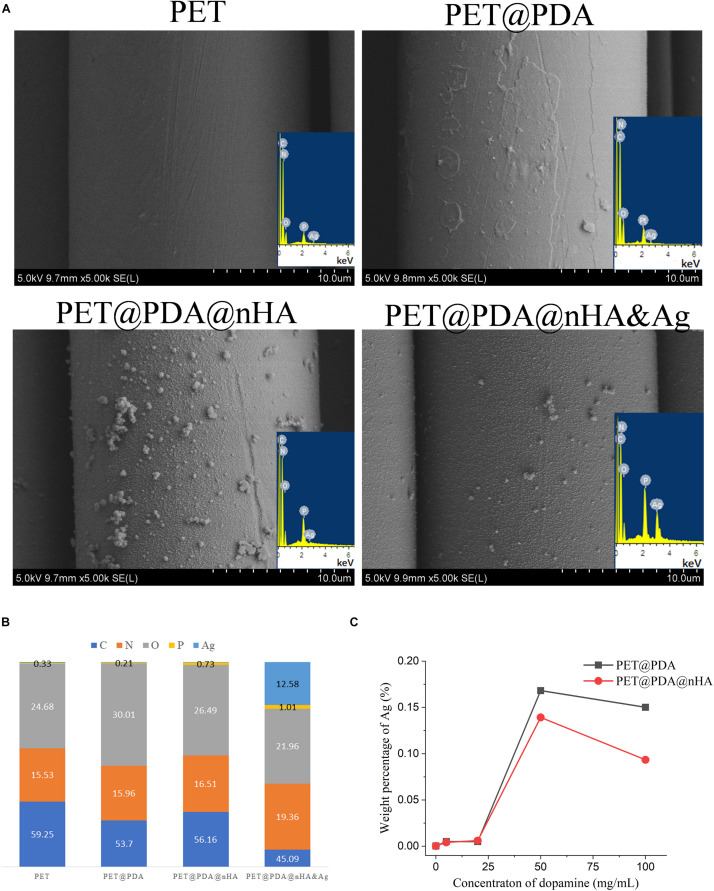
SEM and eds of four PET ligaments **(A)**, weight percentage of five element **(B)** and Ag **(C)** in PET@PDA@Ag and PET@PDA@nHA&Ag ligaments.

Because the surface of PDA is rich in various reductive organic groups, such as amino group, phenolic hydroxyl group and so on, it has been used to reduce metal ions to metal atoms (Wang Z. et al., 2020). When the PET@PDA ligament was placed in a certain concentration of silver nitrate solution, silver ions were first adsorbed on the surface of PDA and then were reduced to silver atoms by PDA. On the other hand, nHA particles bound on the surface of PDA could form insoluble salts with silver ions because of phosphate radicals, which could also adsorb silver ions on nHA. Figure 6A is a scanning electron microscope (SEM) image of PET@PDA@nHA and PET@PDA@nHA&Ag, and the results of energy dispersive spectrometer (EDS). It can be seen from the SEM images that the morphology of PET@PDA@nHA does not change significantly after it reacts with AgNO_3_, and no obvious new nano-structure is produced. However, different results could be seen on the EDS signal. In the figure, no Ag signal is found on PET@PDA@nHA. While there is an obvious Ag peak in the signal of PET@PDA@nHA&Ag, which indicates that although there is no change in morphology, silver atoms had been adsorbed on the surface of PET ligament after reacting with silver nitrate. By comparing the percentage changes of each element (Figure 6B), which was analyzed from EDS data, it can be seen that after nHA adheres to PDA, the signal of phosphorus element with content of about 1% appears in EDS signal, and more than 10% silver element appears after reaction with AgNO_3_. The results of EDS well reflect the adsorption process of nHA and Ag on the surface of PET@PDA artificial ligament in different experimental stages.

At the same time, PDA and nHA may have different reaction abilities with silver ion, which may lead to different Ag^+^ adsorption capacity between PET@PDA and PET@PDA@nHA. In order to verify this inference, PET@PDA and PET@PDA@nHA prepared by the reaction of PET with different concentrations of DA react with silver nitrate solution of the same concentration (concentration of 10 mg/mL). After that, inductively coupled plasma-atomic emission spectrometry (ICP-AES) was used to measure the mass of silver adsorbed on PET with certain mass. The results are shown in Figure 6C. It can be seen from the figure that when the concentration of DA is less than 20 mg/mL, the mass of silver nitrate adsorbed on the surface of two artificial ligaments is rare. When the concentration of DA is greater than 20 mg/mL, a large number of silver atoms can be adsorbed on the surface of PET ligament. The highest adsorption amount appears at the concentration of 50 mg/mL, and the adsorption amount of Ag decreases at higher concentration. The variation of the adsorption capacity may be due to the following reasons: (1) When the concentration of DA is less than 20 mg/mL, hydrogen peroxide tends to oxidize DA completely due to the low concentration of DA in the solution of DA polymerization reaction. As the concentration of DA is higher than 20 mg/mL, the equivalent concentration of hydrogen peroxide is not enough to completely oxidize DA, thus initiating the self-polymerization of DA leading to the formation of a large number of PDA on the PET surface; and (2) When the concentration of DA is high enough, the PDA on the surface of PET would grow too thick due to the fast reaction speed. When Ag^+^ reacts with PDA, part of PDA may fall off from the surface due to oxidation, and the lower layer of PDA without silver atom is exposed, which leads to the relative decrease of Ag content in ICP test. Although the Figure 6C show that PET@PDA@nHA adsorb less Ag atoms, which may be due to the fact that the binding ability of nHA to silver is relatively weak than PDA, it also shows that both PDA and nHA can effectively bind Ag atoms, which provides a good basis for the next step of antibacterial using silver ions.

### Mechanical Property

**FIGURE 7:**
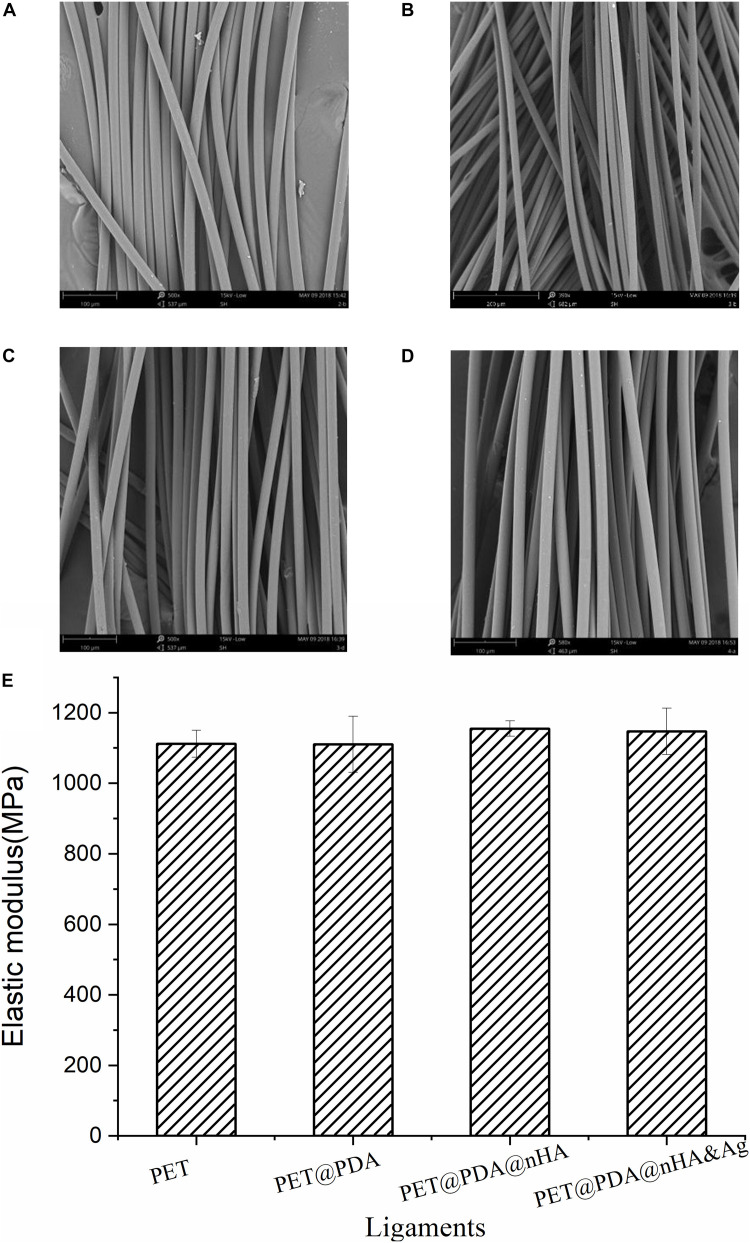
SEM images of PET **(A)**, PET@PDA **(B)**, PET@PDA@nHA **(C)**, and PET@PDA@nHA&Ag **(D)** ligament fibers and **(E)** the elastic modulus of the four ligaments.

One of the reasons that the PET artificial ligament can be used in ACLR are its strong mechanical properties, which can guarantee the patient’s joint can bear daily activity and even a certain intensity after the operation. The mechanical performance of the modified ligaments is a key indicator. Figure 7 enumerates the SEM images and the mechanical properties of the PET, PET@PDA, PET@PDA@nHA (two-step method), and PET@PDA@nHA&Ag artificial ligament. From the figure, we can see that the mechanical properties of the four ligaments have no obvious difference, and the elastic modulus are all maintained at 1,100–1,200 MPa, indicating that the modification process has no negative effect on the main mechanical structure of the fiber.

### Release of Silver Ion and Antibacterial Properties

Ag^+^ could destroy the combination of polysaccharides on the cell wall of bacteria, thus affecting the physiological function of cell wall. At the same time, after entering the pathogen cell, Ag^+^ could combine with the thiol group in the functional protein, which affects the normal proliferation of pathogenic bacteria and leads to the death of pathogenic bacteria. Therefore, the materials that could release silver ions would acquire antibacterial ability. However, the realization of this function requires that silver atoms could be oxidized and dissociated into silver ions to enter the surrounding environment of pathogenic bacteria (Rai et al., 2009). Ag atoms could be oxidized by dissolved oxygen in aqueous solution and further form silver ions dissolved in aqueous solution. At the same time, silver phosphate compounds, as a kind of insoluble salt, could gradually dissociate silver ions in solution, especially under acidic conditions. The synovial fluid in the knee joint can provide a good environment for the release of silver ions, which may provide the basis for the antibacterial PET ligament.

**FIGURE 8:**
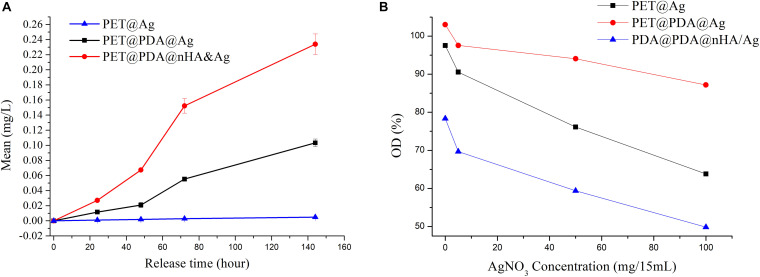
**(A)** The release curve of silver ion of different ligaments, and **(B)** effect of Ag^+^ reaction concentration on antibacterial ability.

In order to understand the release of Ag^+^ from Ag-modified ligaments, PET soaked in Ag^+^ solution (PET@Ag), PET@PDA@Ag, and PET@PDA@nHA&Ag ligaments were placed in normal saline with pH value of 7.4, and the Ag^+^ concentrations were detected when ligaments were soaked in normal saline for different time. Figure 8A shows the release curve of Ag^+^, in which the concentration of DA for PET modified in two ligaments is 50 mg/mL. It can be seen from the figure that the silver ion concentration in the solution of the two ligaments (PET@PDA@Ag and PET@PDA@nHA&Ag) gradually increases with the increase of immersion time in different release time. After soaking for 6 days (144 h), the silver ion concentration in the solution reached 0.22 mg/L. At the same time, it can also be noticed that the artificial ligament modified with nHA has a higher concentration of released Ag^+^, which is related to the higher Ag^+^ release efficiency of silver phosphate complex, which has weak solubility in an aqueous solution. However, compared with the other two ligaments, the Ag^+^ concentration of pure PET was much lower than that of the other two ligaments. The silver ion release curves indicated that both PDA and nHA could form a silver adsorption layer on the PET surface with a sustained release effect.

To analyze the antibacterial ability of three ligaments, AgNO_3_-soaked PET, PET@PDA@Ag, and PET@PDA@nHA&Ag tested against *Staphylococcus aureus*. The results are shown in Figure 8B. As shown in the figure, AgNO_3_-soaked PET, PET@PDA@Ag, and PET@PDA@nHA&Ag artificial ligaments had a certain antibacterial effect after reacting with the AgNO_3_ solution of a certain concentration, which was similar to previous literature reports (Wang K. et al., 2020), and the antibacterial ability increased with the increase of AgNO_3_ reaction concentration, which indicated that the increase of silver ion concentration in the reaction solution had an obvious promoting effect on the antibacterial effect of the artificial ligament. On the other hand, it can also be observed that before the interaction with silver ions, the antibacterial ability of three kinds of ligaments also has an obvious difference. Compared with the control group without any material, the OD value (103%) of a pure PET artificial ligament has no decrease, or even a slight increase, which indicates that the pure PET artificial ligament has no antibacterial property. Compared with the control group, the PET@PDA@nHA ligament has an obvious bacteriostatic effect, and its OD value is 78, while the OD value of the PET@PDA ligament was 98, which means its antibacterial effect was not significant. With the increase of the silver nitrate concentration, the antibacterial properties of all three ligaments were significantly improved. When the concentration of AgNO_3_ in the reaction solution was increased to 6.6 mg/mL, the OD values of PET@Ag, PET@PDA@Ag, and PET@PDA@nHA&Ag were 87, 64, and 50, respectively. The results showed that the antibacterial effect of the materials initiated by silver nitrate could be promoted when the presence of PDA and nHA on the PET surface. The reason for the above phenomenon is that pure PET has a high degree of chemical inertia, which makes it difficult for silver ions to react with PET ligaments. But with the increase of silver ion concentration, its antibacterial effect can be attributed to the effect of electrostatic adsorption and a small amount of silver ions reduced by PET. However, the surface of PET modified by PDA is covered with a reduced layer of DA, which makes the ligaments more capable of reacting with silver ions. For PET@PDA@nHA&Ag, the surface-modified nHA contains a large number of phosphate radicals, and the pKa value of silver phosphate (monohydrate phosphate, dihydrogen phosphate) is relatively high. The formation of these insoluble substances can also promote the sustained release of silver ions in the water.

### Cell Proliferation

**FIGURE 9:**
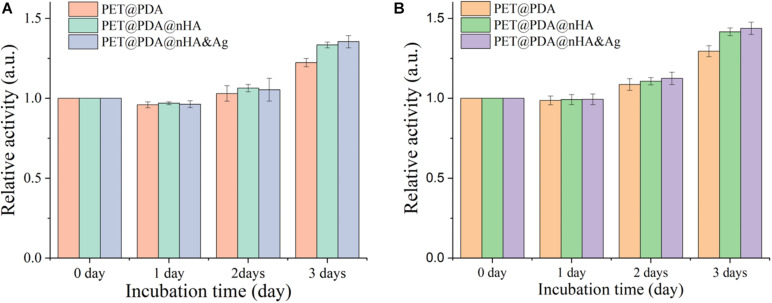
**(A)** NIH3T3 and **(B)** MG-63 cell proliferation of three kinds of ligaments.

Due to nHA and PDA having good biocompatibility, here we use the CCK8 assay to verify whether PET surface-modified PDA and nHA promoted cell proliferation and osseointegration, and whether the introduction of Ag atom brought biological toxicity. The results are shown in Figure 9. From Figure 9, it can be seen that the two cell lines showed similar rules in the process of culture. First, the number of cells in the three modified ligaments on the first day of culture was slightly less than that in pure PET ligaments (relative activity < 1), while the number of cells in the second and third days was significantly higher than that in pure PET ligaments (relative activity > 1). The experimental results show that PDA effectively improves the cell proliferation on the surface of the PET ligament, which may be due to the fact that PDA could effectively change the hydrophobic state of the PET surface (Cai et al., 2018). PDA contains rich amino and hydroxyl groups, which could infiltrate all kinds of nutrients needed for cell proliferation in the water phase on the surface of PET, and amino groups could help the cell to proliferate on the PET surface (Cai et al., 2018). Meanwhile, it also can be seen that nHA-modified ligaments have higher cell activity than PDA-modified ligaments on the second and third day of cell culture. The reason may be that the existence of nHA increases the specific surface area of the PET surface (Lee et al., 2007). nHA, as a major inorganic ceramic material and an essential component of the bone, can further promote the proliferation of osteoblasts on the PET surface. The above factors are the reason why MG-63 osteoblasts in Figure 9 show higher cell activity when cultured with two kinds of ligaments modified with PDA and nHA, (The cell viability of NIH3T3 and MG-63 cultured with PET@PDA@nHA&Ag on the third day was 1.35 and 1.43, respectively), and this result also indicates that the co-existence of PDA and nHA has an add-on effect on the proliferation of osteoblasts. The above results showed that the existence of PDA and nHA could promote the proliferation of cells, and the adsorption of Ag atoms did not significantly affect the growth of cells.

## Conclusion

In this paper, we use the self-polymerization of DA derived from the adhesion principle of mussels, in weak oxidizing and weak alkaline aqueous solutions, and the surface of the PET artificial ligament was successfully modified with PDA. Then, nHA was modified on the surface of PET by using PDA as adhesive layer. TGA showed that the mechanical properties and chemical stability of the PET ligament were not affected by the modification procedure. In the third step, we successfully adsorbed silver atoms on the PET surface by using the reducibility of DA and the phosphate contained in nHA. By testing mechanical tensile, it is found the modification of PDA, nHA, and Ag have no negative effect on the mechanical strength. The results of the silver ion release test showed that the modification of PDA and nHA on the PET surface could provide the sustained release of silver ions into an aqueous solution, and the results of the antibacterial test showed that the PET surface modified with silver atom had significant antibacterial properties. The results of the biological activity test showed that both PDA and nHA could significantly enhance the cell proliferation; meanwhile, the presence of Ag atoms did not significantly affect the growth of cells. On the other hand, this study also faces some challenges in the further clinical application, such as how to adapt to the tissue growth transition between the bone tunnel and joint cavity in the process of tendon–bone healing, and so on. In summary, this modification method of nHA and silver atom based on PDA provides a new insight to further improve the biocompatibility and expand the function of the PET artificial ligament.

## Data Availability Statement

The raw data supporting the conclusions of this article will be made available by the authors, without undue reservation.

## Author Contributions

All authors listed have made a substantial, direct and intellectual contribution to the work, and approved it for publication.

## Conflict of Interest

The authors declare that the research was conducted in the absence of any commercial or financial relationships that could be construed as a potential conflict of interest.
