# Supplementary material for: Preparation and Properties of Antibacterial Polydopamine and Nano-Hydroxyapatite Modified Polyethylene Terephthalate Artificial Ligament
Source: Front Bioeng Biotechnol. 2021 Mar 31;9:630745. doi: 10.3389/fbioe.2021.630745 (PMC8044552; doi:10.3389/fbioe.2021.630745)
Supplement: Supplementary file 1 [file Data_Sheet_1.docx]

Supplementary Material

## Supplementary Figures


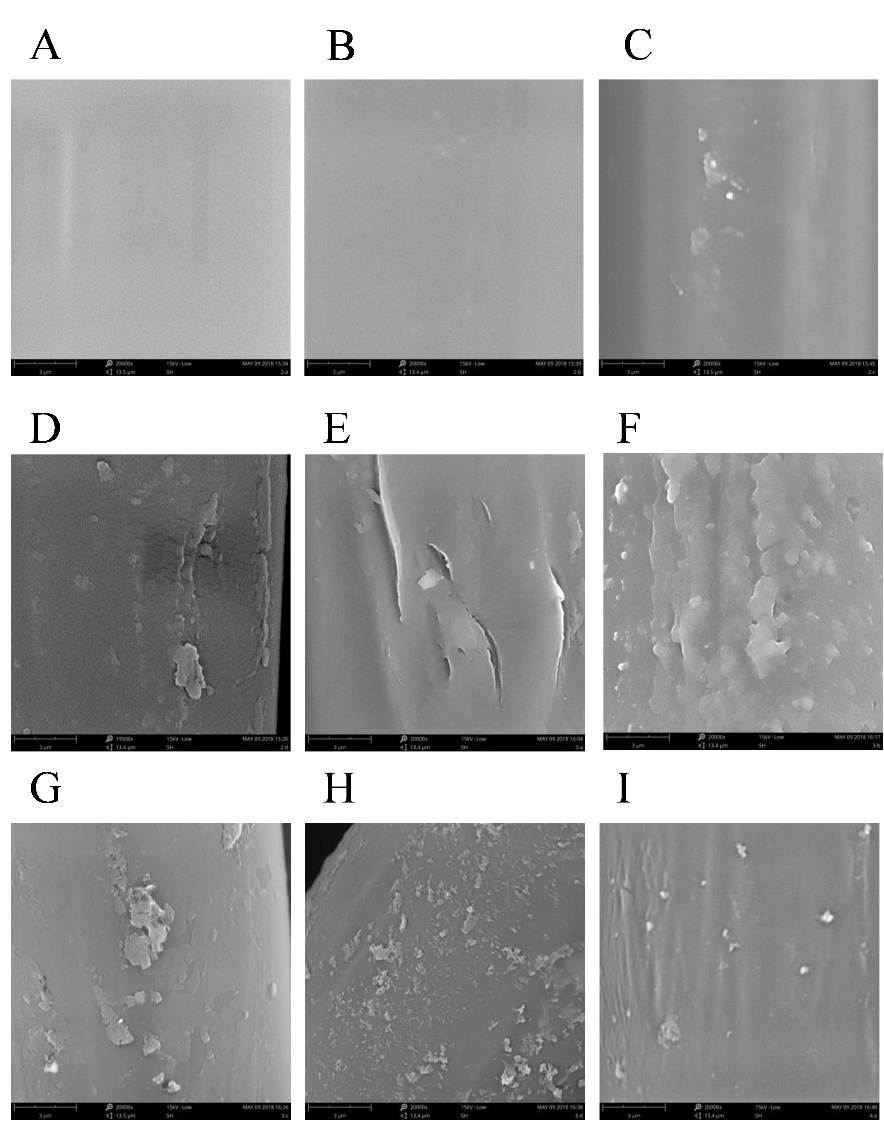


**Supplementary Figure 1.** TEM images of different concentration of dopamine modified PET ligament surface. (a) pure PET, (b)1mg, (c)5mg, (d)10mg, (e)20mg, (f)50mg, (g)100mg. And nHA modified PET@PDA ligament surface(h) and PDA and nHA simultaneously modified PET ligament surface (i)


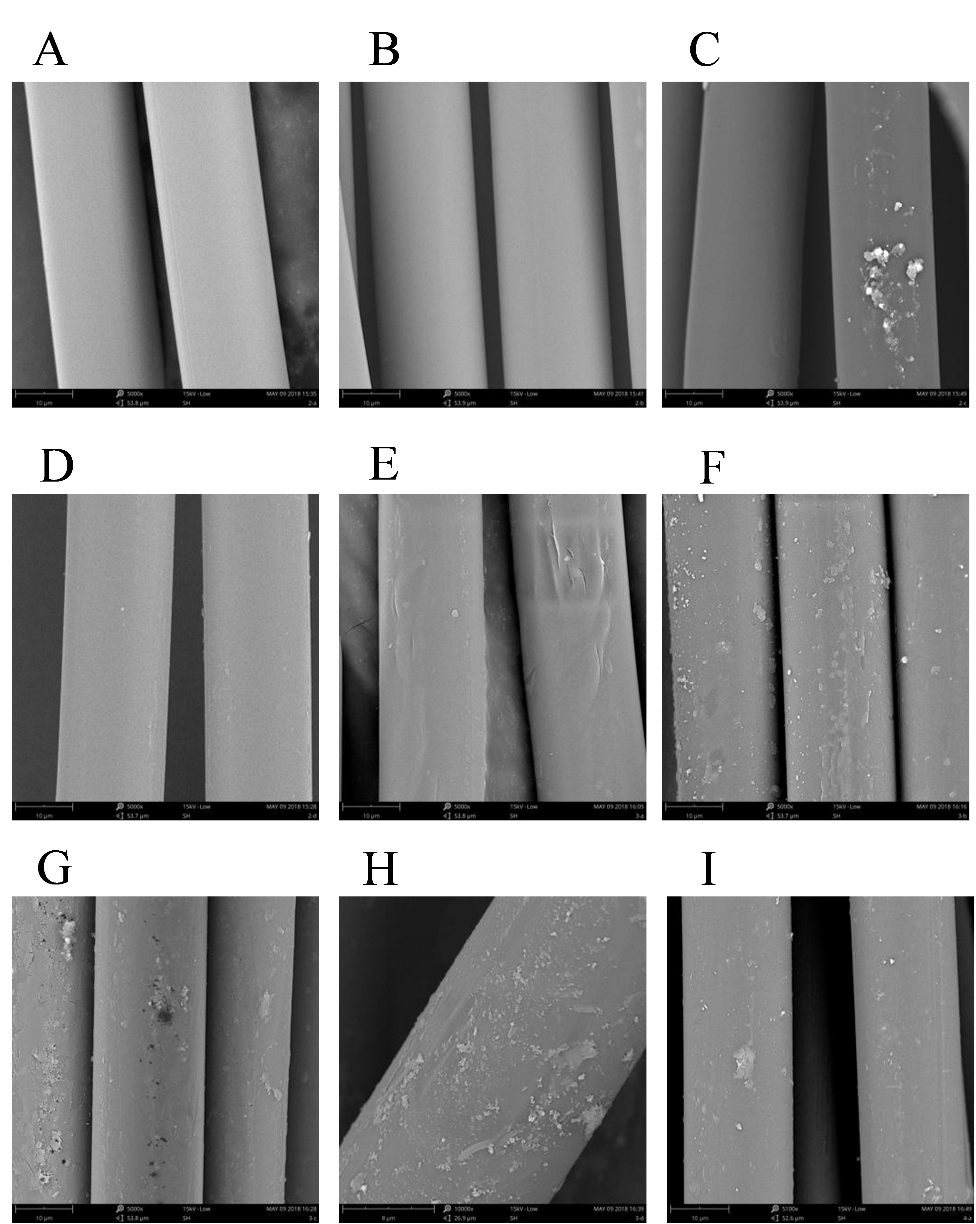


**Supplementary Figure 2.** TEM images of different concentration of dopamine modified PET ligament surface. (a) pure PET, (b)1mg, (c)5mg, (d)10mg, (e)20mg, (f)50mg, (g)100mg. And nHA modified PET@PDA ligament surface(h) and PDA and nHA simultaneously modified PET ligament surface (i).
